# Landscape-level variation in spring leaf phenology is driven by precipitation seasonality in the Mexican red oak *Quercus castanea*

**DOI:** 10.1093/aobpla/plae067

**Published:** 2024-12-09

**Authors:** Tamara C Ochoa-Alvarez, Gonzalo Contreras-Negrete, Libny Ingrid Lara-De La Cruz, Antonio González-Rodríguez

**Affiliations:** Facultad de Biología, Universidad Michoacana de San Nicolás de Hidalgo (UMSNH), Edificio “R” Planta Baja, Ciudad Universitaria Francisco J. Múgica S/N, Morelia, Michoacán 58030, México; Ciencias Agrogenómicas, Escuela Nacional de Estudios Superiores Unidad León, Universidad Nacional Autónoma de México (UNAM), León, Guanajuato, 37684, México; Instituto de Geología, Universidad Nacional Autónoma de México (UNAM), Circuito Exterior S/N, Coyoacán, C.U., Ciudad de México 04510, México; Instituto de Investigaciones en Ecosistemas y Sustentabilidad, Universidad Nacional Autónoma de México (UNAM), Antigua Carr. a Pátzcuaro 8701, Morelia 58190, México

**Keywords:** Adaptation, Cuitzeo basin, environmental heterogeneity, phenotypic plasticity, leaf functional traits

## Abstract

Water availability is one of the essential factors that determine the distribution of plant species, as well as their ecological strategies. The study of leaf phenology, in conjunction with other leaf traits of an ecological nature, such as functional traits, makes it possible to determine the life history strategies of plant species and their variation along environmental gradients, which in turn influences the demographic rates of populations. In the present study, we analysed the effect of water availability at the landscape scale on spring leaf phenology and foliar traits such as leaf mass per area (LMA) and leaf thickness (LT) in the oak species *Quercus castanea* from a tropical latitude in central-western Mexico. Six sites were selected in the Cuitzeo basin, Michoacán, across a water availability gradient, ranging from 766 mm to 1145 mm of mean annual precipitation. Leaf samples were collected from 10 adult trees at each site and LT and LMA were estimated. Leaf phenology was monitored for each tree every two weeks between March and July for two consecutive years, 2021 and 2022, alongside soil moisture measurements. Temperature and precipitation variables for the two study years were obtained from meteorological stations and long-term bioclimatic variables from the Worldclim database. Significant spatial and temporal variation in leaf phenology was observed. Earlier leaf development and shorter development times were observed with increased soil moisture in March and April, and with higher precipitation in October of the previous year. Also, sites with long-term higher precipitation seasonality and with lower precipitation of the warmest quarter showed longer development times. A positive association between development times and leaf thickness was also observed. *Quercus castanea* shows a brevideciduous leaf phenology but with significant variation among populations, reflecting spatiotemporal mosaics of environmental and genetic variation and in covariation with leaf functional traits such as leaf thickness.

## Introduction

Plant phenology refers to the periodic production of vegetative and reproductive structures; particularly, to the timing of initiation and the duration of growth and reproductive cycles (Schwartz 2003). The analysis of leaf phenology has taken great relevance in recent years, since it is considered an essential adaptive trait in plants (Cleland *et al.*, 2007; Gerst *et al.*, 2017; Armstrong and Greenwood 2021). That is, through the course of their evolution, plants have optimized the period of the year that is most favourable for leaf burst and leaf expansion under different environments. Such adjustment allows plants to meet their metabolic requirements while avoiding damage due to unfavourable environmental conditions, as extreme temperatures, or periods of drought (Singh *et al.*, 2017). Therefore, leaf phenology plays a fundamental role in mediating carbon balance and herbivory levels (Polgar and Primack, 2011; Cleland *et al.*, 2012; Gallinat *et al.*, 2015).

Different environmental cues are related to leaf phenology patterns in plants, varying according to ecosystem type. In temperate deciduous forests, the increase in temperature during the spring triggers the sprouting of leaves of most tree species (Chuine and Régnire 2017). When temperature decreases, as it does when autumn starts, the process of senescence and leaf fall begins (Borchert *et al.*, 2005). Another important factor linked to leaf phenology is photoperiod (Schaber and Badeck 2003; Samtani *et al*.,, 2015). This is because a minimum daily exposure to sunlight is essential to accomplish plant biological functions and, therefore, the time of leaf growth is usually associated to daylength. In a future climate, photoperiod could be important in limiting the timing of spring phenology when increasingly warmer climatic conditions accelerate development (earlier bud break). These changes in phenology will tend to be observed mostly in temperate latitudes where there is less adaptation to high temperatures with respect to tropical latitudes (Basler and Körner, 2012; Dewan *et al.*, 2020; Faticov *et al.*, 2020; Koenig *et al.*, 2021).

In contrast, in other biomes which also experience a marked environmental seasonality, such as tropical deciduous forests, leaf phenology is mainly determined by the temporal variation in precipitation, while temperature variation is of less importance (Armstrong and Greenwood 2021). However, in tropical regions with large seasonal variation in precipitation, phenology cannot be predicted from climatic data alone. This is because the onset of leaf growth is mainly determined by seasonal variation in tree water status, day length and old leaf fall (Borchert 1994, Borchert 2004). The growing season in these forests is not significantly reduced even during prolonged periods of low rainfall, since water tends to remain stored in the soil, protecting trees against seasonal drought (Borchert *et al.*, 2005).

The genus *Quercus*, with more than 500 extant species, is one of the most important groups of woody plants in the northern hemisphere and often a dominant component of very valuable and diverse ecosystems, such as forests and shrubland communities (Hipp *et al.*, 2018; Cavender-Bares 2016). In general, compared to other forests, oak forests are characterized by greater species diversity, stratification, litter production and soil fertility (Bargali *et al.*, 2015). *Quercus* species have been the basis of numerous studies seeking to explain how climatic cues influence phenological patterns. Research has focussed mainly on the influence of temperature and precipitation patterns on leaf phenology of species in temperate zones (Morin *et al.*, 2009; Vitasse *et al.*, 2009; Basler and Körner 2012). For example, higher temperatures can induce early bud burst and leaf flushing in Californian oaks such as *Q. lobata* and *Q. agrifolia*, whereas eastern oaks as *Q. rubra* and *Q. alba* are more responsive to spatial and temporal variations in precipitation, because the deep tap roots of *Q. agrifolia* and *Q. lobata* may protect them from temporal variation in precipitation (Gerst *et al.*, 2017). However, in another study with five Californian oak species, *Q. agrifolia*, *Q. kelloggii*, *Q. douglasii*, *Q. garryana* and *Q. lobata*, precipitation in the winter season was found to be the main climatic driver of spring leaf phenology (Armstrong-Herniman and Greenwood 2021). Studies in Europe have focussed on analysing the influence that temperature increase resulting from global change has had as the main driver of phenological changes in oaks on that continent (Morin *et al*, 2010; Dantec *et al*, 2014; Caignard *et al*, 2017; Journé *et al*, 2021). For example, the number of dry spells, the number of frost days in the autumn and the spring, and the number of hot days in the autumn were associated with longer canopy duration in *Q. robur* and *Q. petraea*, with consequences for reproductive output (Journé *et al.*, 2021).

Studies of leaf phenology in oak species at tropical latitudes are scarcer and have shown contrasting responses. For example, significant correlations were found of the timing and duration of leaf development with temperature and soil water potential for *Q. magnoliifolia*, but for *Q. resinosa* the duration of leaf development was negatively correlated with precipitation, with both species growing at different positions of the same altitudinal gradient (Hernández-Calderón *et al.* 2013). These results therefore indicate different drivers for leaf phenology in these two partially coexisting oak species and suggest that temperature, precipitation and soil water availability are relevant to understand leaf phenology of oaks in tropical regions.

In this study, we focussed on leaf phenology variation of the Mexican red oak *Q. castanea* at the landscape level in the Cuitzeo basin, Michoacán. From casual observations, the species is considered deciduous or brevideciduous (Aguilar-Romero *et al.*, 2017; Kaproth *et al.*, 2023) but there are no formal studies in this regard. The basin has an area of approximately 4000 km^2^ and is highly heterogeneous topographically and climatically, with an increase in precipitation and a decrease in temperature from north to south and with elevation (Mendoza *et al.*, 2006). Among approximately 13 oak species that occur in this basin, *Q. castanea* is the most abundant and widely distributed, and it can be found in diverse communities such as oak forests, pine-oak forests and subtropical scrublands (Aguilar-Romero *et al.*, 2016; Lara-De la Cruz *et al*., 2020). Previous studies identified a significant association between leaf thickness (a trait indicating the degree of sclerophylly) of *Q. castanea* populations in the basin and precipitation seasonality (Lara-De la Cruz *et al*., 2020). In tropical oaks such as *Q. oleoides*, an association between phenological patterns (i.e. degree of leaf deciduousness) and leaf mass per area has been found (Cavender-Bares and Ramírez-Valiente 2017), highlighting the importance of simultaneously considering phenology and other functional traits for understanding population adaptation to environmental gradients within oak species.

Here, we hypothesized that *Q. castanea* populations located in areas with higher precipitation seasonality in the Cuitzeo basin will be more deciduous than those in sites with lower seasonality. To test the hypothesis, we determined foliar phenology patterns of *Q. castanea* at six sites along the climatic gradient in the basin over a two-year period. Then, we assessed the influence of climatic and environmental variables by obtaining data of temperature and precipitation as well as soil moisture. In particular, we evaluated the following questions: (i) Is there temporal (i.e. between years) and spatial (i.e. among sites) variability in leaf phenology patterns of *Q. castanea* across the climatic gradient? (ii) What are the main environmental variables that influence leaf phenology variation? (iii) How does variation in leaf functional traits (leaf thickness and leaf mass per area) relate to leaf phenology?

## Materials and Methods

### Study system

The study was carried out in the Cuitzeo basin, Michoacán. In this area, populations of *Q. castanea* can be found between 2000 and 2800 meters above sea level (Herrera-Arroyo *et al.*, 2013). The basin is located between 19° 30ʹ 0″ and 20° 0ʹ 0″ latitude N and 100° 45ʹ 0″ and 101° 30ʹ 0″ longitude W, with an area of about 4000 km^2^. It is highly heterogeneous in climate, topography and vegetation (Leal-Nares *et al.*, 2010; Lara-De la Cruz *et al.*, 2020).

### Sampling design

**Figure 1.**
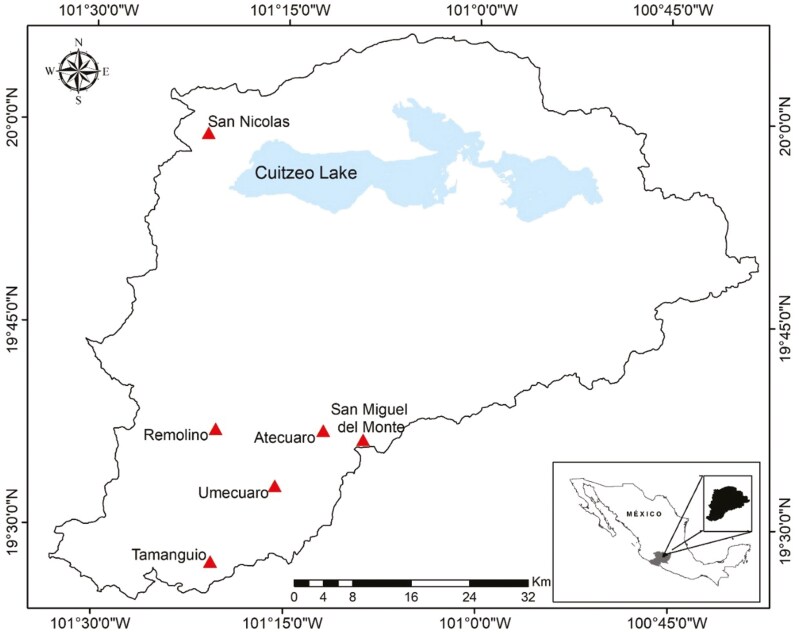
Map of *Quercus castanea* populations studied in the Cuitzeo basin.

Six sites with populations of *Quercus castanea* were selected across the geographic and climatic gradient of the Cuitzeo basin. According to WorldClim (https://www.worldclim.org/) data for the 1970–2000 period, annual precipitation in the six sites ranges from 909 mm to 1299 mm and mean annual temperature from 13.8°C to 16.9°C (**Table 1**, **Fig. 1**). In each of the six sites chosen, 10 adult *Q. castanea* trees with diameters at breast height between 30 and 80 cm were randomly selected (maintaining a minimum distance of at least 20 m between individuals) and marked in order to keep a record of their leaf phenology during the years 2021 and 2022.

**Table 1.** *Quercus castanea* sites used for phenological monitoring and collection. Precipitation and temperature data were retrieved from the WORDLCLIM (https://www.worldclim.org/) database.

Site (Abbreviation)	Latitude	Longitude	Elevation (masl)	Annual Precipitation (mm)	Mean annual temperature (°C)
**Tamanguío (TA)**	19.98°	−101.35°	2457	1299	13.8
**Remolino (REM)**	19.55°	−101.26°	2523	1181	14.2
**Atécuaro (AT)**	19.61°	−101.15°	2317	1060	15.5
**San Miguel (SM)**	19.62°	−101.2°	2097	1096	15.3
**Umécuaro (UM)**	19.62°	−101.34°	2116	945	16.9
**San Nicolás (SN)**	19.45°	−101.34°	2233	909	16

### Leaf phenology

**Figure 2.**
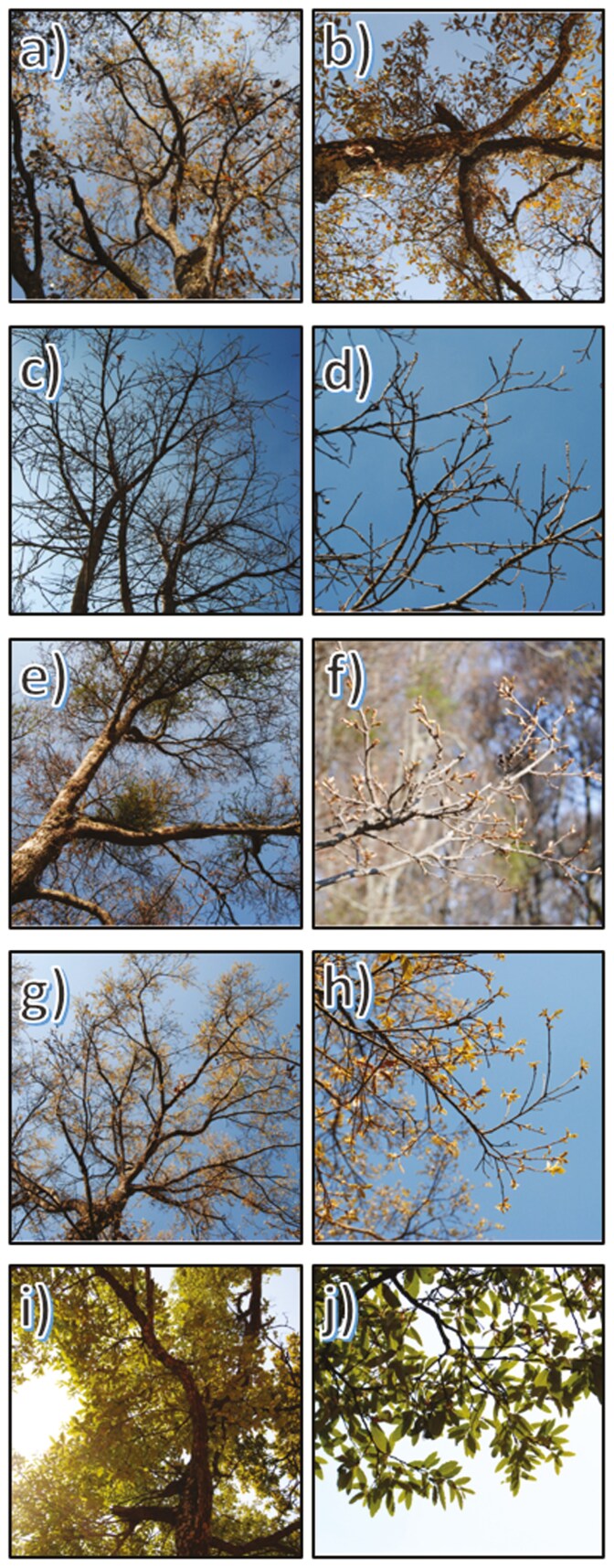
Stages of leaf phenology of *Quercus castanea.* (**A)** Foliage of the previous year (tree crown); (**B)** Foliage of the previous year (branch); (**C)** dormant buds (tree crown); (**D)** dormant buds (branch); (**E)** bud burst (tree crown); (**F)** bud burst (branch); (**G)** leaf unfolding (tree crown); (**H)** leaf unfolding (branch); (**I)** developed leaves (tree crown) and (**J)** developed leaves (branch).

Since previous studies indicated that the period of highest litterfall production in *Q. castanea* in the Cuitzeo basin occurs between February and March (Chávez-Vergara *et al.*, 2015), monitoring was conducted every two weeks in the period from March to July of 2021 and 2022. During visits, previously marked individuals were monitored, for which photographs were taken of the branches and crowns of the trees, to later determine the stage of leaf development of each tree. Five leaf development stages were determined and assigned a numerical value for subsequent analysis. The stages were as follows: foliage of the previous year = 0, dormant buds = 1, bud burst = 2, leaf unfolding = 3, and developed leaves = 4 (**Fig. 2**).

### Meteorological and soil moisture data

Temperature (minimum, mean and maximum) and rainfall monthly data were obtained from 40 weather stations of the National Water Commission (Comisión Nacional del Agua) (CONAGUA 2020) located in the northern part of Michoacán state, near the study sites (**see**Supporting Information—**Fig. S1**) for 2021 and 2022. To obtain specific data for the six study sites, we performed regionalized spatial interpolation of precipitation and temperature from weather stations with the R package RegRAIN (version 0.1.0) (Alzate-Velásquez *et al.*, 2017). This package incorporates multiple linear regression, spline and inverse distance weighting interpolations. For calculations the program uses a digital elevation model, as well as climate data. In our case, a digital elevation model for Michoacán state, with resolution of 15 m, was obtained from the National Institute of Statistics and Geography (Instituto Nacional de Estadística y Geografía, INEGI). Raster maps were obtained from the interpolations and monthly minimum, mean and maximum temperature, and precipitation, were obtained for the six study sites using ArcGIS ver. 10.3.

In addition, we used WordlClim data, which describe average temperature and precipitation data for a 30-year period (1970–2000). We downloaded the 19 bioclimatic variables for each monitored site (https://www.worldclim.org/). Subsequently, the climatic variables that were redundant (with correlation higher than 0.7) were eliminated, leaving a total of eight variables with which the regression analyses with the phenological variables were carried out. The variables used were the following: mean annual temperature; isothermality (defined as mean diurnal range of the temperature divided by the temperature annual range and multiplied by 100); mean temperature of the driest quarter; mean temperature of the warmest quarter; annual precipitation; precipitation seasonality (PS), which measures the deviation from a uniform distribution of rainfall throughout the year, with small values indicating little or no seasonal variation in precipitation; (Walsh and Lawler 1981); precipitation of the driest quarter (PDQ); and precipitation of the warmest quarter (PWQ). Besides, during each visit to the study sites, we measured soil moisture near the base of the trunk of each monitored individual at a depth of approximately 15 cm, using the soil moisture Delta-T Device (Cambridge, UK) sensor.

### Leaf functional traits

For each monitored *Q. castanea* individual we measured dry leaf thickness (LT) and leaf mass per area (LMA). For this purpose, 15 mature autumn leaves were collected in November from each monitored tree during the two study years. Leaves were pressed and dried at 40 °C for 2 days. Leaf thickness was then measured using a digital Vernier device. The weight of each of the 15 leaves collected for each tree was obtained on an analytical balance and leaf area was measured in the ImageJ program using digitialized images. LMA was obtained by dividing the dry mass (weight) by the leaf area.

### Data analysis

To analyse leaf phenology patterns of each monitored tree during 2021 and 2022, dose–response relationships were modelled using the ‘drc’ library in R (Ritz *et al.*, 2015). In these analyses, the independent variable was the day of the year (DOY) of each phenological observation, considering as Day 1 January first, and the dependent variable was the leaf development stage of each individual. Data obtained for both years were separately analysed. First, a four-parameter log-logistic model (i.e. lower limit, slope, ED50, upper limit,) was tested. Then, based on this model, we tested the best fitting model (i.e. L.L.2, 3, 4 or 5 parameters, linear, quadratic, cubic and Weibull 1 or 2) which was selected according to the Akaike Information Criterion (AIC) with the ‘mselec’ function. From these models, for each individual tree we estimated ED50 (effective dose-response at 50%, which in our case represents the number of days that elapse from the beginning of the year until the day on which the tree reaches 50% of its leaf development, DOY). Additionally, we calculated development time (DT), as the number of days elapsed between stage 1 (dormant buds) and stage 4 (fully expanded leaves).

Non-parametric Kruskall–Wallis tests were performed to determine if there were differences in ED50 and DT between populations within a single year and between years for each population, using values for each individual tree.

To identify environmental variables influencing leaf phenology variation, we used data of the eight selected bioclimatic variables obtained from the Worldclim database and performed regression analysis with each of the phenological variables (ED50 and DT). Similarly, we used the monthly averages of maximum, minimum and mean temperature as well as monthly rainfall for the years 2021 and 2022. Finally, soil moisture data registered during each monitoring date was also used as an independent variable

To determine the association between leaf functional traits and phenological variables, linear regression analyses were performed with data of LT and LMA obtained for each year and corresponding values of ED50 and DT.

## Results

### Spatial and temporal variation in leaf phenology

**Figure 3.**
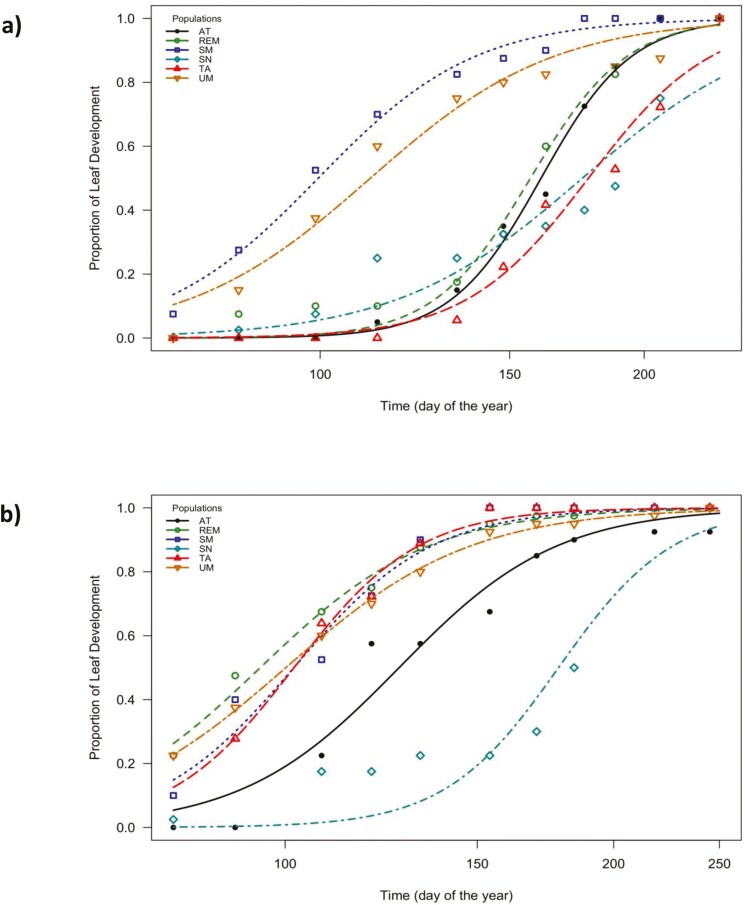
Log-logistic models for leaf development of *Q. castanea* in six sites of the Cuitzeo basin in (A) 2021 and (B) 2022. See Table 1 for site abbreviations.

There were significant differences among populations within years and for populations between years for ED50 and DT (**Table 2**, **Fig. 3**). In 2021, the population average DOY for ED50 varied between 104 and 179, corresponding to San Miguel and Tamanguío, respectively. In 2022, the DOY for ED50 varied between 95 and 177, with San Miguel and Remolino showing the first value and San Nicolás showing the later value. Between years, the ED50 for Atécuaro, Remolino and Tamanguío were significantly different, with 2022 showing an earlier DOY in the three cases.

**Table 2.** Differences in mean phenological variables (ED50 and development time) among monitored sites and between years 2021 and 2022. Values highlighted in black correspond to those that showed significant differences between years according to a Kruskal–Wallis test. Different letters indicate significant differences between populations in the same year. The numbers in parentheses correspond to the standard error. The abbreviations correspond to the names of the populations as shown in Table 1.

Site	ED50 (doy)		Development time (number of days)	
	2021	2022	2021	2022
**TA**	**179 (7.2)** ^a^	**100 (19.7)** ^ab^	**74 (4.9)** ^b^	**38 (4.9)** ^c^
**REM**	**163 (6.9)** ^a^	**95 (18.6)** ^b^	80 (9.6) ^b^	56 (9.6) ^bc^
**AT**	**163 (6.9)** ^a^	**124 (18.6)** ^ab^	64 (4.6) ^bc^	77 (4.6) ^ab^
**SM**	104 (6.9) ^b^	95 (18.6) ^b^	37 (3.2) ^c^	42 (3.2) ^bc^
**UM**	117 (6.9) ^b^	120 (18.6) ^ab^	67 (13.4) ^bc^	72 (13.4) ^abc^
**SN**	175 (6.9) ^a^	177 (18.6) ^a^	128 (9.4) ^a^	108 (9.4) ^a^

**doy, day of the year**.

For the year 2021, the shortest DT was observed in San Miguel (37 days), and the longer in San Nicolás (128 days). In 2022, Tamanguío showed the shortest development time (38 days) and San Nicolás the longest (108). Differences between years were only significant in the case of Tamanguío, with a decrease from 74 days in 2021 to 38 days in 2022.

As expected from the observed interannual variation in ED50 and DT, individual tree values of these two variables were significantly correlated (*P* < 0.01) between years 2021 and 2022, but with low or moderate R^2^ (0.15 for ED50 and 0.41 for DT) (**[**see Supporting Information—**Fig. S2**).

### Correlations between phenological and environmental variables

**Figure 4.**
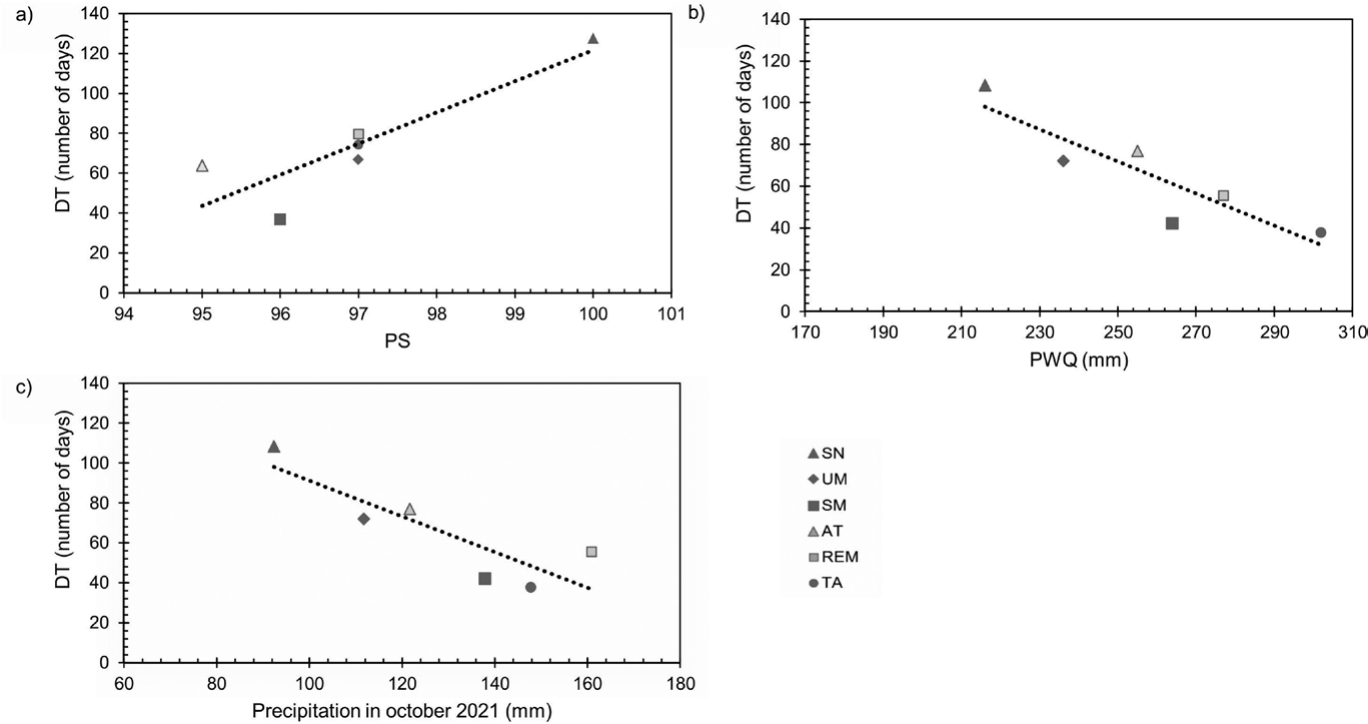
Linear regression analyses for significant relationships between mean population values of leaf phenology variables (ED50 and development time, DT) and environmental variables. (A) DT in 2021 and PS (*R*^2^ = 0.76; *P* = 0.02). (B) DT of 2022 and PWQ (*R*^2^ = 0.79; *P* = 0.02). (C) DT of 2022 in and precipitation in October 2021 (*R*^2^ = 0.73; *P* = 0.03).

In Fig. 4 we show only the correlations that were significant between population mean phenological variables for 2021 and 2022 and the long-term bioclimatic variables and the precipitation variables from 2021 and 2022 derived from meteorological stations. DT of 2021 was positively correlated with the average PS for 1970–2000 (*R*^2^ = 0.76; *P* = 0.02) (Fig. 4a), and DT of 2022 was negatively correlated with average PWQ for 1970–2000 (*R*^2^ = 0.79; *P* = 0.02) (Fig. 4b). Additionally, DT of 2022 was negatively correlated with the precipitation observed in October 2021 in the sites (*R*^2^ = 0.73; *P* = 0.03) (Fig. 4c), showing that the amount of rainfall at the end of the rainy season of the previous year may influence leaf phenology of the following year.

**Figure 5.**
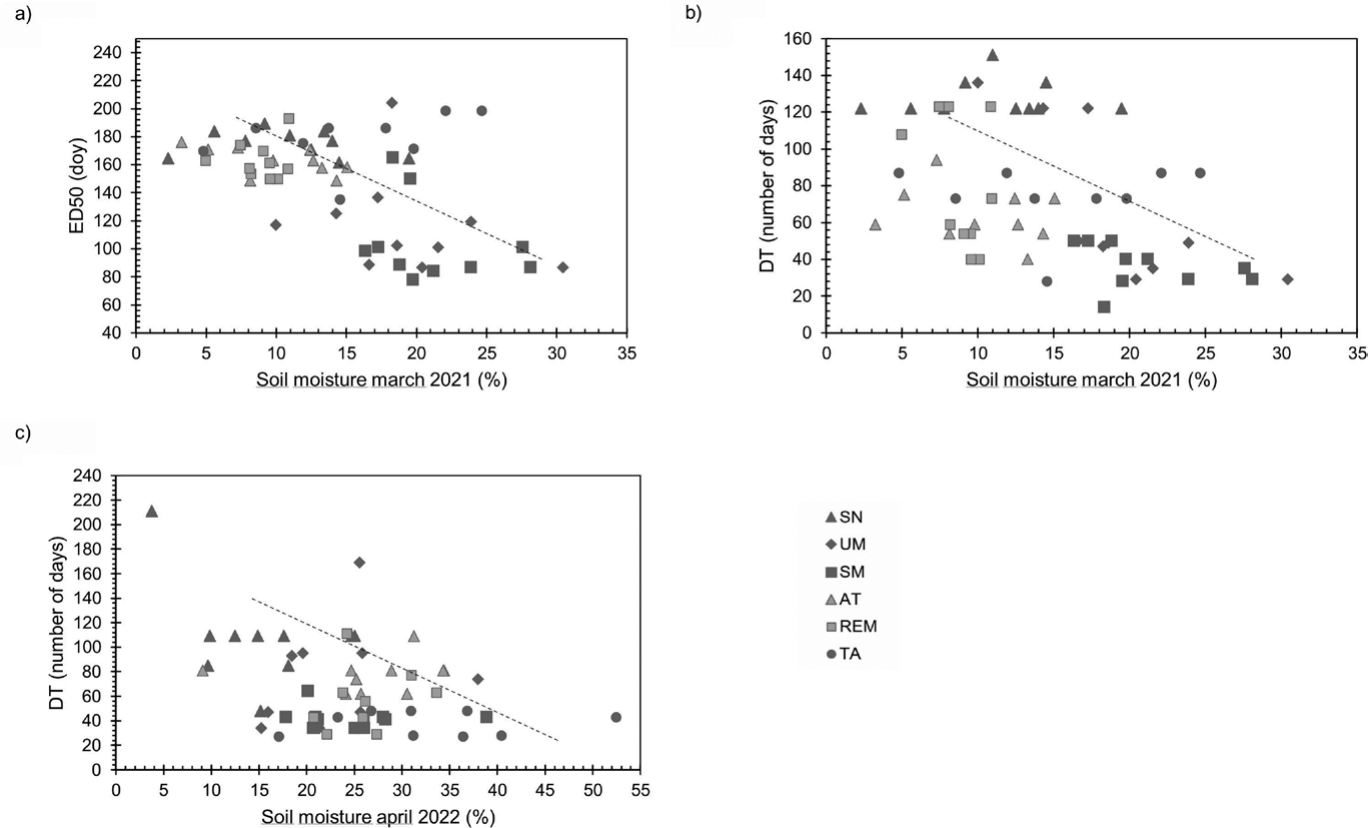
Linear regression analysis for significant relationships between soil moisture by date monitored and phenological variables of individual trees. (A) ED50 of 2021 and soil moisture in March 2021 (*R*^2^ = 0.29; *P* < 0.0001). (B) DT in 2021 and soil moisture in March 2021 (*R*^2^ = 0.22; *P* = 0.0002). (C) DT in 2022 and soil moisture in April 2022 (*R*^2^ = 0.13; *P* = 0.0053).

Using values for individual trees, we found that the ED50 and DT of 2021 were negatively correlated with soil moisture in March of the same year (*R*^2^ = 0.29; *P* < 0.0001 and *R*^2^ = 0.22; *P* = 0.0002, respectively) (Fig. 5A and B). In 2022, DT was negatively correlated with soil moisture in April of the same year (*R*^2^ = 0.13; *P* = 0.0053) (**Fig. 5C**).

### Phenological stage with respect to soil humidity

In order to determine the soil moisture at the time when bud burst occurs, we plotted the average monthly soil moisture by population for the two study years with the values corresponding to the phenological stage observed on each date. In 2021 (**see Supporting Information—****Figure S3**), the change from stage 1 to stage 2 in all the populations occurred when soil humidity was between 10% and 20%. The values of soil humidity for all monitored dates ranged between 4% and 40%. In 2022 (**[see Supporting Information—****Figure S4**), in San Nicolás and Atécuaro, bud burst occurred when soil humidity was between 20% and 35%, while in Umécuaro, El Remolino, San Miguel and Tamanguío bud burst occurred with 5–10% of soil humidity.

### Relation of functional variables with leaf phenology

**Figure 6.**
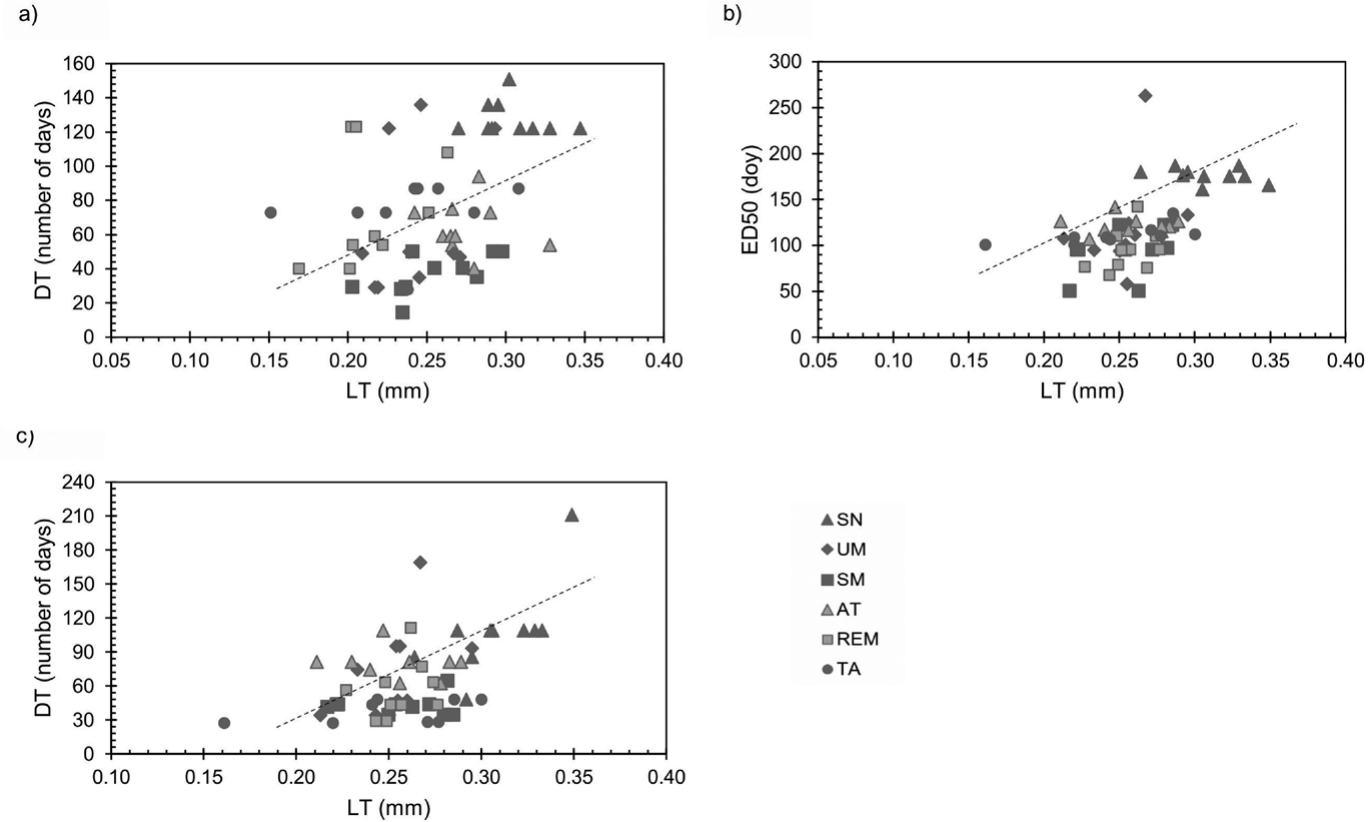
Linear regression analysis for significant relationships between leaf functional traits (LT in mature fall leaves) and phenological variables of individual trees. (A) DT in 2021 (*R*^2^ = 0.13; *P* = 0.005). (B) ED50 in 2022 (*R*^2^ = 0.29; *P* = 0.0001). (C) DT in 2022 (*R*^2^ = 0.29; P = 0.0001).

In 2021, with individual tree data, we observed a positive correlation between DT and LT (*R*^2^ = 0.13; *P* = 0.005) (**Fig. 6**) and in 2022 both ED50 (*R*^2^ = 0.29; *P* < 0.0001) and DT (*R*^2^ = 0.29; *P* < 0.0001) were correlated with LT. We did not find correlations between phenological variables and LMA.

## Discussion

The results of our study clearly indicate that there is significant spatial and temporal variation in the spring leaf phenology of *Q. castanea* populations in the Cuitzeo basin, despite the short geographic distance among sampling sites, but probably associated to the considerable environmental heterogeneity in the area (Mendoza *et al.* 2011; Maldonado-López et. al, 2015). In a previous study in the Cuitzeo basin, water use strategies were compared among nine oak species, including *Q. castanea*, along an aridity gradient (Aguilar-Romero *et al.* 2017). The study found that species occurring in the more arid parts of the basin tend to be more deciduous than species in less arid areas. *Q. castanea* was characterized as a brevideciduous species with comparatively intermediate values of xylem resistance to embolism and an intermediate position in the aridity gradient (Aguilar-Romero *et al.*, 2017). Our data from this study also confirm that *Q. castanea* shows a brevideciduous leaf phenology, as defined by Singh and Kushwaha (2005). According to this classification, species with periods of less than 4 months without leaves are considered brevideciduous and those with more than 4 months without leaves are considered deciduous.

We showed that the average difference in ED50 between the population with the earlier leaf development and the one with the later development was on average 78.5 days in the two study years, and that the duration of the period elapsed between the dormant buds and fully expanded leaves varied from 37 to 128 days. Also, from one year to the next, three populations differed in ED50, with an earlier DOY by an average of 62 days in 2022 in comparison to 2021. DT was also significantly shorter in one population in 2022, with a reduction from 74 to 38 days. We found that two of the most significant variables to which the observed variation in spring leaf phenology was related to were fall precipitation of the previous year (October) and soil moisture in the spring of the same year (March or April). Interestingly, the month of October was unusually rainy in 2021, with an average precipitation of 128.7 mm across the six sites and a range between 92.3 and 160.8 mm, in comparison to 15.7 mm (8.9–21.4 mm) in 2020 and 38 mm (24.3–45.8 mm) in 2022. Based on the significant correlation between DT in 2022 and precipitation in October of 2021, we consider that the phenological advancement observed in 2022 was related to the increased water availability resulting from these exceptional fall rains, suggesting the existence of lagged effects. Besides this, for both years, we observed that the timing and intensity of the first spring rains are very important for triggering leaf development through their influence on soil humidity.

Additional associations were identified between phenological parameters and the long-term mean values of two bioclimatic variables, PS and PWQ. For our six study sites, PS ranged from 95 to 100, indicating that the overall rainfall regime is markedly seasonal with a long dry season (Walsh and Lawler 1981). Thus, all these patterns point to the critical importance of water availability during the driest period of the year for the phenological patterns of *Q. castanea*, similar to what occurs in seasonally dry tropical forest trees (Borchert *et. al*., 2005) occurring at lower elevations in the state of Michoacán and much of western Mexico.

Several provenance tests, common garden experiments, and more recently genomic studies, have identified high heritability values and signatures of local adaptation in the leaf phenological variation patterns among populations of various tree species (Vitasse *et al.,* 2009; Albert *et al.,* 2011; Papper and Ackerly 2021; Wright *et al.*, 2021; Meger *et al.,* 2024). In *Q. castanea* such studies are still lacking, but our results suggest that the phenological variation we observed is reflecting spatiotemporal mosaics of environmental and genetic variation (Blonder *et al.*, 2023). The correlations between DT and long-term averages of PS and PWQ could have arisen as a result of differential selection pressures at each population, given high environmental heterogeneity despite short geographic distances, and resulting in adaptive variation; but this hypothesis requires formal evaluation. On the other hand, the interannual phenological differences and the moderate correlation of individual ED50 and DT values between years point out to a role for plastic responses in *Q. castanea* trees adjustment to the prevailing conditions during the initiation of leaf development and also of lagged effects of previous months, or even years.

Our study is also useful to understand the evolution of the genus *Quercus* in the Mexican territory. As oaks migrated and diversified into the Mexican landscapes, they adapted mostly to moisture gradients (Hipp *et al.,* 2018), with temperature, and particularly minimum temperatures, imposing less significant selection pressures in this process. Also, oaks in Mexico and Mesoamerica exhibited a doubling in transitions among leaf habitat states (i.e. deciduousness, brevideciduousness and evergreeness) in comparison to USA counterparts, suggesting the evolutionary importance of this trait, together with other ecophysiological and functional responses, in the adaptation to various levels of drought stress (Hipp *et al.,* 2018; Kaproth *et al.,* 2023). Concurrently, in our results, at the intraspecific level, temperature variables did not contribute to explain phenological differences among *Q. castanea* populations, with only precipitation and soil humidity variables being significant, while in oaks from higher latitudes, temperature variables have been recurrently found to be important for explaining phenological variation among populations (Papper and Ackerly 2021; Journé *et al.,* 2021; Wright *et al.,* 2021; Meger *et al.,* 2024).

In general, we observed that the percentage of soil moisture required to start leaf development was 15%, revealing that the accumulated water in the soil is an environmental cue that initiates leaf burst. However, in some populations, the soil moisture did not reach this percentage and despite this, bud burst occurred. This means that some trees are making use of their water reserves or accessing deep underground water. From studies that have been conducted on *Quercus* species, it is known that these trees use deep roots to efficiently absorb available water to survive severe droughts (Gieger and Thomas 2002; Suseela and Tharayil 2020).

*Q. castanea* is probably not only using the environmental signal of the onset of the rainy season, but also seems to be making use of other factors that indicate the adequate time for leaf development. Other cues or drivers that influence leaf phenology may be irradiance (Saleska *et al.*, 2007) and photoperiod (Basler and Körner 2012), although these may vary in response to environmental constraints (Tang *et al.*, 2016). In addition to abiotic cues, there are also biological factors such as competition, herbivory, resource limitation, and genetics, which control phenology (Wolkovich *et al.,* 2014).

On the other hand, we observed that some foliar functional traits, such as leaf thickness, are associated to the phenological variation of *Q. castanea*. In particular, thicker leaves were associated to longer development times and later ED50. Leaf thickness exhibits associations with various anatomical and physiological features of the leaves and its functional significance has been discussed based on large scale surveys across many tree species from all major biomes (Niinemets 1999; Niinemets 2001). In general, LMA is positively related to LT, and also to leaf density, but the latter two are not necessarily correlated, suggesting independent control over these two variables through different mechanisms. Higher photosynthetic efficiency has been observed as leaf thickness increases, resulting in shorter ‘leaf pay-back’ times, that is, the time required to assimilate the amount of carbon necessary for leaf construction (Niinemets 2001). It is possible that in our study system, the higher deciduousness and thicker leaves observed in *Q. castanea* individuals from sites with more seasonal precipitation arises from a trade-off between shorter canopy duration because of the water availability restrictions imposed by a more marked dry season, and the need of a higher photosynthetic efficiency to compensate carbon investment in this comparatively shorter period. However, this hypothesis would require assessment, together with alternative hypothesis (i. e. thicker leaves may be more resistant to herbivory or to other stress factors).

In conclusion, we observed that the populations of *Q. castanea* found in sites with lower water availability and with a more marked seasonality of precipitation in the Cuitzeo basin have a slower leaf growth compared to the less seasonal populations. Leaf phenology differed between sites, both for 2021 and 2022, which means that such variation is responding to environmental changes. The oaks of Michoacán seem to adjust their phenology according to precipitation and water accumulation in the environment, a behaviour similar to that of seasonally dry forest species. We conclude that, in addition to environmental signals, plant-specific foliar traits, such as LT, play a crucial role in the phenology of oaks at tropical latitudes.

## Supporting Information

The following additional information is available in the online version of this article –

**Figure S1.** Weather stations in the state of Michoacán near the Cuitzeo basin.

**Figure S2.** Linear regression analysis for significant relationships between DT in 2021 and 2022 and ED50 in 2021 and 2022 of individual trees. (a) DT in 2021 and 2022 (*R*^2^ = 0.41; *P* < 0.0001). (b) ED50 in 2021 and 2022 (*R*^2^ = 0.15; *P* = 0.0028).

**Figure S3.** Phenological stages corresponding to six populations of *Quercus castanea* with their soil moisture. Dates in 2021.

**Figure S4.** Phenological stages corresponding to six populations of *Quercus castanea* with their soil moisture. Dates in 2022.

plae067_suppl_Supplementary_Materials

## Data Availability

Data are available in a repository and can be accessed via a DOI link. The data underlying this article are available in the Harvard Dataverse Repository, at https://doi.org/10.7910/DVN/ENHAMD.
