# Supplementary material for: Landscape-level variation in spring leaf phenology is driven by precipitation seasonality in the Mexican red oak Quercus castanea
Source: AoB Plants. 2024 Dec 9;17(2):plae067. doi: 10.1093/aobpla/plae067 (PMC12038158; doi:10.1093/aobpla/plae067)
Supplement: plae067_suppl_Supplementary_Materials [file plae067_suppl_supplementary_materials.pdf]

SUPPORTING INFORMATION

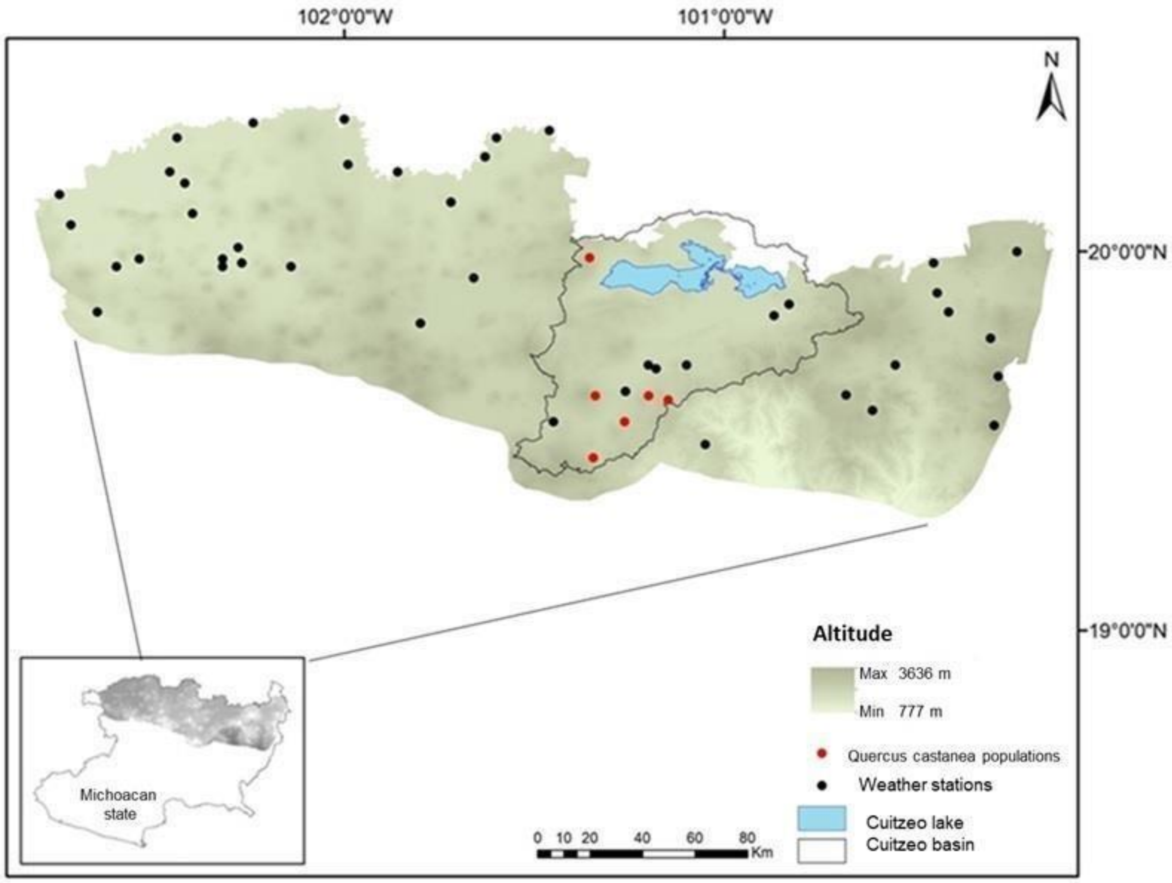

Figure S1. Weather stations in the state of Michoacán near the Cuitzeo basin.

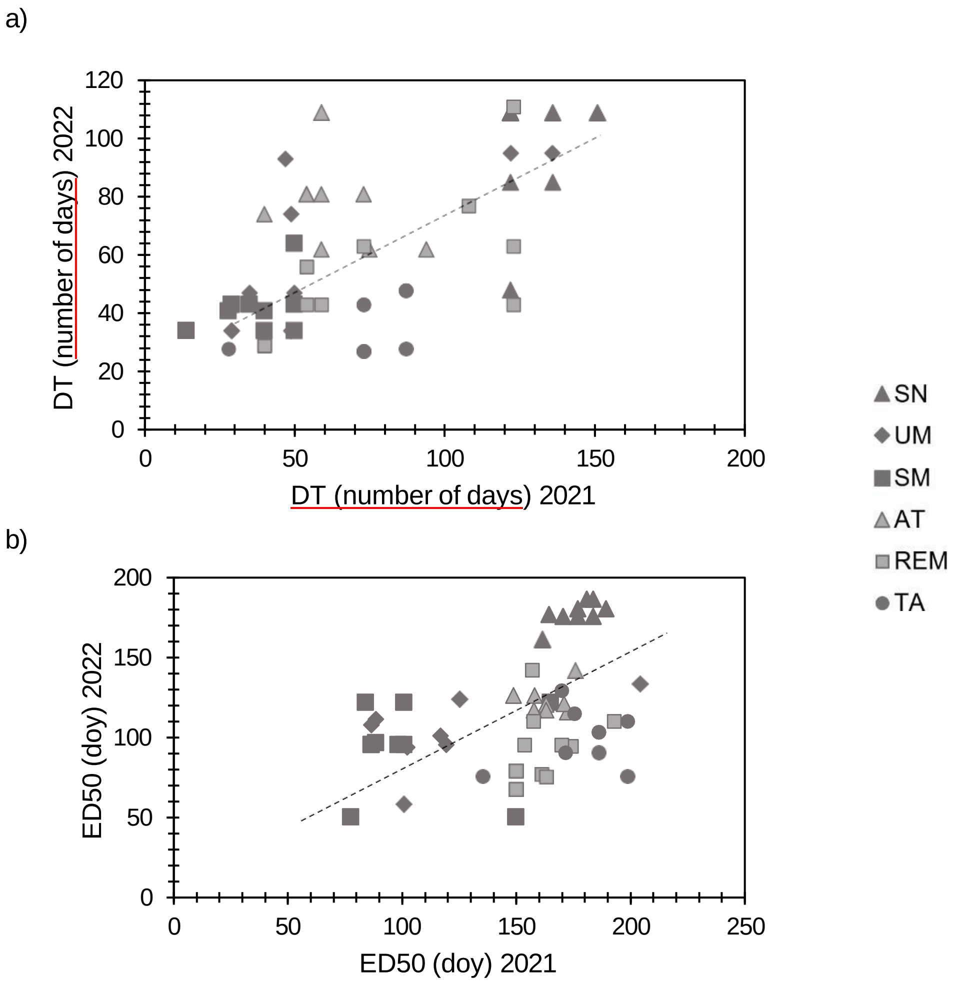

Figure S2. Linear regression analysis for significant relationships between DT in 2021 and 2022 and ED50 in 2021 and 2022 of individual trees. a) DT in 2021 and 2022 ($R^2 = 0.41$; $P < 0.0001$). b) ED50 in 2021 and 2022 ($R^2 = 0.15$; $P = 0.0028$).

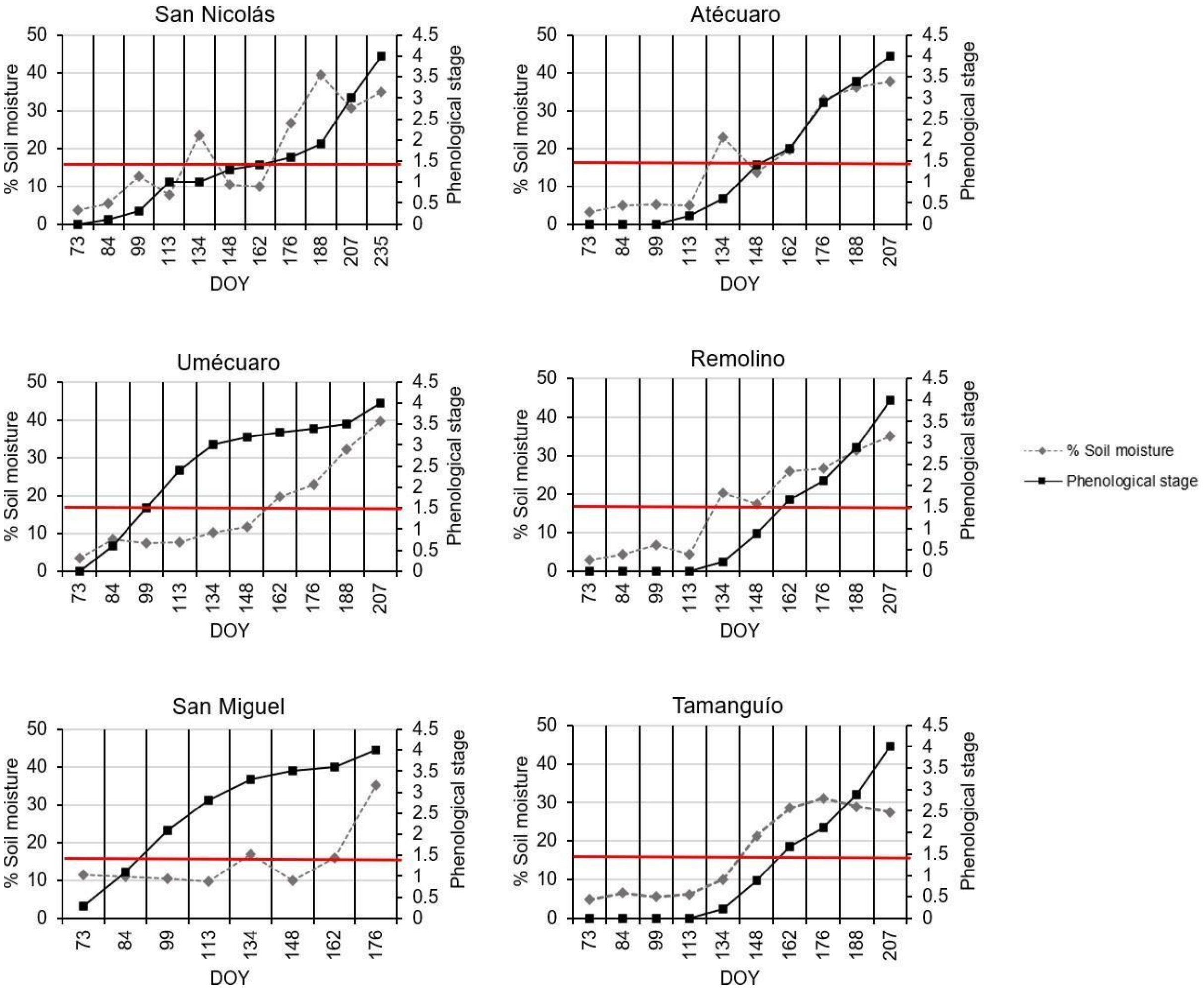

Figure S3. Phenological stages corresponding to 6 populations of *Quercus castanea* with their soil moisture. Dates in 2021.

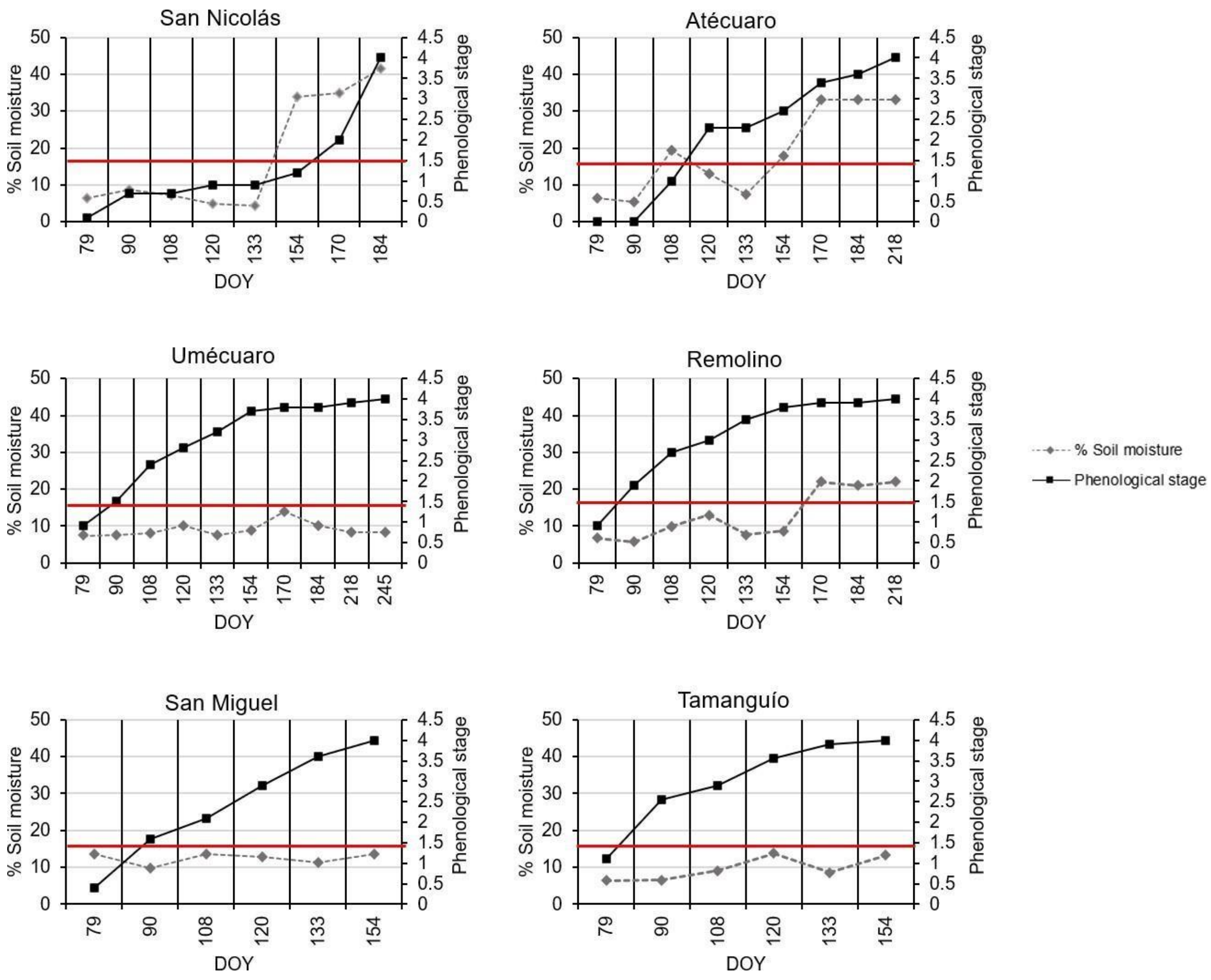

Figure S4. Phenological stages corresponding to 6 populations of *Quercus castanea* with their soil moisture. Dates in 2022.
